# Clinical performance of 3 commercially available point-of-care antibody tests for *Brucella canis* in dogs

**DOI:** 10.1093/jvimsj/aalag073

**Published:** 2026-04-30

**Authors:** Callum Atkins, Megan Madden, Shauni Emanuel, Ioannis Oikonomidis, Rebecca Franklin-Guild, Cassandra Guarino, Glynn Woods

**Affiliations:** Hospital for Small Animals, Royal (Dick) School of Veterinary Studies, University of Edinburgh, Roslin, Edinburgh, EH25 9RG, United Kingdom; Hospital for Small Animals, Royal (Dick) School of Veterinary Studies, University of Edinburgh, Roslin, Edinburgh, EH25 9RG, United Kingdom; Hospital for Small Animals, Royal (Dick) School of Veterinary Studies, University of Edinburgh, Roslin, Edinburgh, EH25 9RG, United Kingdom; Department of Veterinary Anatomy, Physiology and Pathology, Institute of Infection, Veterinary and Ecological Sciences, University of Liverpool, Leahurst Campus, Liverpool, CH64 7TE, United Kingdom; Department of Population Medicine and Diagnostic Sciences, College of Veterinary Medicine, Cornell University, Ithaca, NY 14853, United States; Department of Population Medicine and Diagnostic Sciences, College of Veterinary Medicine, Cornell University, Ithaca, NY 14853, United States; William R. Pritchard Veterinary Medical Teaching Hospital, School of Veterinary Medicine, University of California Davis, Davis, CA 95616, United States

**Keywords:** *Brucella canis*, canine brucellosis, zoonotic disease, lateral flow assay, diagnostic accuracy

## Abstract

**Background:**

Brucellosis in dogs, caused by *Brucella canis*, is a zoonotic disease often underdiagnosed because of diagnostic challenges and subclinical infection. Bacterial culture, the reference standard, is impractical and has limited sensitivity because of intermittent or low-grade bacteremia. Accurate point-of-care (POC) testing could facilitate timely management and infection control.

**Hypothesis/Objectives:**

Evaluate the diagnostic performance of 3 POC lateral flow assays for detection of *B. canis* antibodies in dogs, using the Canine Brucella Multiplex (CBM) assay as the reference serologic assay.

**Animals:**

Fifty serum samples from 50 client-owned dogs submitted for B. canis serology.

**Methods:**

Samples were classified as CBM-positive (*n* = 10; culture-positive dogs) or CBM-negative (*n* = 40; healthy dogs undergoing export testing). Assays were run in duplicate and independently interpreted by 2 observers. Positive percent agreement (PPA), negative percent agreement (NPA), and inter-reader agreement were calculated relative to CBM.

**Results:**

The Anigen Rapid C.Brucella Ab assay demonstrated 90.0% PPA (95% CI, 55.5-99.8) and 100% NPA (95% CI, 91.1-100). The FASTest® *BRUCELLA canis* assay demonstrated 100% PPA (95% CI, 69.1-100) and 92.5% NPA (95% CI, 79.6-98.4). The Canine Brucella LPS Antibody Rapid Test demonstrated 0% PPA (95% CI, 0-30.9) and 97.5% NPA (95% CI, 86.8-99.9). Inter-reader agreement was almost perfect for all assays (κ = 0.81-1.00).

**Conclusions and clinical importance:**

The Anigen Rapid C.Brucella Ab and FASTest® *BRUCELLA canis* assays showed good agreement with the CBM assay and may be useful rule out tests in dogs with clinical signs compatible with brucellosis. In low prevalence settings, positive results require confirmatory testing, and false negatives remain possible.

## Introduction

Brucellosis in dogs, caused by *Brucella canis*, a small, gram-negative, non-spore-forming aerobic coccobacillus, is a globally distributed zoonotic disease.^1–3^ Transmission occurs through direct contact with infected fluids, venereal and vertical routes, and exposure via aerosolization of infected materials.^1^^,^^2^ Infected dogs may shed bacteria for prolonged periods, posing a risk to humans, particularly in kennels and veterinary settings, and to vulnerable groups such as pregnant and immunocompromised individuals.^3–6^

The global spread of *B. canis* has been facilitated by increased international dog movement and the lack of regulated pre-import testing, with outbreaks in previously non-endemic countries raising concern for both canine and human health.^5–9^ Mandatory pre-import testing has now been introduced in the United Kingdom for dogs imported from Romania, but reliable point-of-care (POC) assays remain critical for identifying infected animals already in the country and in regions where similar measures are not yet in place.^10^

Clinical signs in dogs are variable and often non-specific, including reproductive failure, lymphadenopathy, uveitis, and discospondylitis.^1–3^^,^^11^ Subclinical infections are common, further complicating detection and management.^1–3^^,^^12^^,^^13^ The highest zoonotic risk arises from contact with reproductive fluids or aborted fetuses from infected dogs.^2^^,^^3^ Infections in humans, although rare, typically manifest as with mild, flu-like illness but may result in severe complications such as endocarditis and osteomyelitis.^1^

Diagnosis of brucellosis is challenging. Conventional methods include bacterial culture, rapid slide agglutination tests (RSAT), agar gel immunodiffusion (AGID) and polymerase chain reaction (PCR).^1^^,^^8^^,^^9^^,^^12^^,^^13^ Blood culture is considered the antemortem reference standard for diagnosing *B. canis* infection, but sensitivity is highly stage- and sample-dependent and decreases in chronic or subclinical disease because of intermittent bacteremia and tissue sequestration.^8^^,^^9^^,^^12^^,^^13^ Cultures from urine, semen, or reproductive tissues may increase yield in selected cases.^8^^,^^12^^,^^13^ Serologic assays may cross-react with other gram-negative bacteria and PCR can produce false-negative results depending on disease stage.^1^^,^^13–15^ The Canine Brucella Multiplex (CBM) assay, which utilizes a multiplex fluorescent bead-based platform (Luminex) with capture reagents consisting of recombinant *B. canis*-specific proteins, including BP26 and a peptide from Omp31, is increasingly used to support diagnosis and disease monitoring.^16–19^ The CBM assay is used as a quantitative screening serologic test, with non-negative results typically confirmed using a reference serologic panel (2-mercaptoethanol RSAT [2ME-RSAT] and AGID II), interpreted in parallel. In a recent study, the 2-antigen CBM assay had an estimated sensitivity of 91.6% (95% credible interval: 85.2%-98.0%) and specificity of 94.9% (95% credible interval: 92.3%-96.9%) relative to the reference panel.^19^ The CBM also generates quantitative antigen-specific values that may support serial monitoring of infected dogs, with published evidence demonstrating associations between decreasing titers and clinical resolution and between persistent or rebounding titers and relapse.^17^^,^^19^

Reliance on specialized laboratories for culture and CBM emphasizes the urgent need for POC diagnostic tests that provide timely patient-side results, enabling immediate clinical decision-making, targeted antimicrobial treatment, and effective infection control. Rapid in-clinic diagnosis supports prompt implementation of isolation measures, protects staff health, decreases the operational and psychological burden associated with delayed or uncertain results, and minimizes unnecessary treatments or euthanasia based on presumptive diagnoses. Given the potential clinical, ethical, and public health consequences of a false positive result, particularly in low prevalence settings, high assay specificity is particularly important for POC screening for *B. canis* infection.

Several POC lateral flow assays (POC-LFAs) for *B. canis* are commercially available and widely used in the United Kingdom. Independent evaluation of POC assays is needed to determine diagnostic performance compared to the serologic reference standard.^15^ We aimed to evaluate and compare the diagnostic accuracy of 3 commercially available POC-LFAs—Anigen Rapid C.Brucella Ab (BioNote, Inc., Korea), Canine Brucella LPS Antibody Rapid Test (Rapid Labs Ltd., United Kingdom) and FASTest® *BRUCELLA canis* (Diagnostik Megacor, Austria)—using sera from dogs with confirmed *B. canis* infection and from confirmed negative controls. We hypothesized that assays incorporating *B. canis*-specific recombinant antigens would demonstrate superior diagnostic agreement compared with those based on less specific antigen targets. Given the absence of a definitive reference standard for brucellosis in dogs, diagnostic performance in our study is reported as positive percent agreement (PPA) and negative percent agreement (NPA) relative to comparative serologic testing (CBM) rather than sensitivity and specificity.

## Materials and methods

Serum samples from 50 client-owned dogs in the United States with known *B. canis* serologic results were obtained from specimens submitted for routine diagnostic testing to the Animal Health Diagnostic Center (AHDC), Cornell University (Ithaca, NY, United States; Table 1).

**Table 1:** Comparison of the Cornell University AHDC reference assays and of the 3 commercially available POC-LFAs used for testing the 50 samples (10 CBM-positive and 40 CBM-negative).

Assay		CBM-positive dogs (*n* = 10); positive, *n* (%)	CBM-positive dogs (*n* = 10); negative, *n* (%)	CBM-negative dogs (*n* = 40); positive, *n* (%)	CBM-negative dogs (*n* = 40); Negative, *n* (%)
**Reference**	*B. canis* culture	10 (100)	0 (0)	Not performed	Not performed
	CBM	10 (100)	0 (0)	0 (0)	40 (100)
	AGID II	9 (90)	1 (10)	0 (0)	40 (100)
	2ME-RSAT	10 (100)	0 (0)	0 (0)	40 (100)
**Test**	Anigen Rapid C.Brucella Ab	9 (90)	1 (10)	0 (0)	40 (100)
	Canine Brucella LPS Antibody Rapid Test	0 (0)	10 (100)	1 (2.5)	39 (97.5)
	FASTest® *BRUCELLA canis*	10 (100)	0 (0)	3 (7.5)	37 (92.5)

Table 1 summarizes the results for all reference assays and POC-LFAs stratified by CBM classification (CBM-positive, *n* = 10; CBM-negative, *n* = 40). Each sample was tested in duplicate for each POC-LFA for quality control; agreement statistics were calculated using a single dog-level classification per assay. All CBM-positive dogs were culture positive.

Abbreviations: 2ME-RSAT = 2-mercaptoethanol rapid slide agglutination test; Ab = antibody; AGID II = agar gel immunodiffusion test using cytoplasmic antigen; CBM = Canine Brucella Multiplex; LPS = lipopolysaccharide; *n* = number; POC-LFAs = point-of-care lateral flow assays.

Samples were collected from dogs with confirmed *B. canis* infection based on positive bacterial culture and concordant serology and from clinically healthy dogs that were negative on all reference serologic assays. Reference testing at AHDC included the 2ME-RSAT, AGID II, and the CBM assay, performed as previously described.^19^ Positive samples (*n* = 10) were from animals with a concurrent or recent (within the preceding month) *B. canis*-positive culture result; all were CBM-positive. Clinical signs were recorded for most of these culture-confirmed cases, although detailed clinical histories were not evaluated for our study. Negative samples (*n* = 40) were from clinically healthy dogs undergoing pre-export *B. canis* screening to meet international movement requirements; all were negative on both the CBM assay and the combined 2ME-RSAT/AGID II reference panel. Submission for *B. canis* testing was at the discretion of the attending clinician.

After completion of diagnostic testing, serum samples were stored at −20 °C at Cornell University AHDC. Samples with sufficient residual volume and meeting the above inclusion criteria were selected, comprising 40 negative and 10 positive sera in accordance with the American Society of Veterinary Clinical Pathology (ASVCP) guidelines for method comparison studies.^20^ Samples were shipped on dry ice, maintained at −20 °C, and tested between September 2024 and March 2025. The POC-LFAs were performed at 2 centers: Hospital for Small Animals (HfSA), University of Edinburgh (Edinburgh, United Kingdom) and AHDC, Cornell University (Ithaca, NY, United States). To minimize handling of potentially infectious material, CBM-positive sera were tested at AHDC and CBM-negative sera were tested at HfSA.

Three commercially available POC-LFAs were evaluated: Anigen Rapid C.Brucella Ab (BioNote, Inc., Korea), Canine Brucella LPS Antibody Rapid Test (Rapid Labs Ltd., United Kingdom) and FASTest® *BRUCELLA canis* (Diagnostik Megacor, Austria). All assays were performed using serum samples only; EDTA whole blood and plasma were not used. All assays were performed according to manufacturer instructions (Table 2). Briefly, for each assay, the test cassette, reagents, and serum were allowed to equilibrate to room temperature (15-30 °C). The cassette was placed on a horizontal surface and the manufacturer-recommended volume of serum was added to the sample well followed by the corresponding volume of supplied assay diluent. After incubation for the specified time at room temperature, results were read immediately. Tests were considered valid only if a positive control line was visible at the recommended time point.

**Table 2:** Summary of the 3 commercially available POC-LFA kits used, their rules of interpretation and reported accuracy.

Assay	Specimen	Volume (μL)	Time (min)	Rules for interpretation	Reported accuracy^a^
Anigen Rapid C.Brucella Ab	whole blood plasma serum	10	10-25	positive result: the 2 color bands (T and C) within the result window negative result: one C line appears in the result window	accuracy: ≥ 90.0% (compared to 2ME-RSAT)
Canine Brucella LPS Antibody Rapid Test	whole blood plasma serum	5	5-10	positive result: the presence of both C line and T line, no matter T line is clear or vague negative result: only clear C line appears	sensitivity: 97.5% specificity: 96.7% (compared to RT-PCR method)
FASTest® *BRUCELLA canis*	whole blood plasma serum	25	20	positive result: a pink-purple T line of any intensity and a pink-purple C line appear negative result: only pink-purple C line appears	sensitivity 72.7% specificity 90.7% (compared to Rose Bengal Test)

Table 2 summarizes the specific testing requirements, rules for interpretation and reported accuracy of 3 commercially available POC-LFAs.

^a^Reported accuracy as documented on manufacturer leaflet included with assay. Abbreviations: 2ME-RSAT = 2-mercaptoethanol rapid slide agglutination test; Ab = antibody; C = control; LPS = lipopolysaccharide; POC-LFAs = point-of-care lateral flow assays; RT-PCR = reverse transcription-polymerase chain reaction; T = test.

All assays were conducted in duplicate and interpreted independently by 2 operators who, because of biosafety protocols and the zoonotic risk of *B. canis*, were not blinded to CBM classification. Results were determined according to manufacturer instructions (Table 2) and consensus interpretations were compared to CBM results. In cases of disagreement between the 2 primary readers, a third operator (also not blinded to CBM classification) reviewed the test result, and the majority interpretation was recorded as the final result. Duplicate testing was performed for quality control purposes and diagnostic agreement was assessed at the dog level rather than the test level. For each assay, a dog was classified as POC-positive only if both replicate tests were positive and POC-negative only if both replicate tests were negative. Discordant replicate results were conservatively classified as incorrect (false-negative for CBM-positive dogs and false-positive for CBM-negative dogs) to avoid overestimation of diagnostic performance. Ethical approval was obtained from Veterinary Ethical Review Committee (VERC), University of Edinburgh (VERC Reference: 142.23).

Positive percent agreement (PPA) and negative percent agreement (NPA), with corresponding 95% CIs, were calculated for each POC-LFA using CBM classification as the reference serologic assay (positive, *n* = 10; negative, *n* = 40). Because CBM is a comparative serologic method rather than a definitive gold standard, agreement statistics (PPA and NPA) are reported instead of sensitivity and specificity. Youden indices and 95% CIs were calculated as a measure of overall discriminatory performance for each assay. Inter-reader agreement between the 2 primary operators was assessed using Cohen’s kappa coefficient (κ), with values interpreted as slight (0.01-0.20), fair (0.21-0.40), moderate (0.41-0.60), substantial (0.61-0.80), or almost perfect (>0.80). Statistical analyses were performed using MedCalc® Statistical Software version 23.3.7 (MedCalc Software Ltd, Ostend, Belgium; https://www.medcalc.org; 2025).

## Results

Fifty serum samples (10 CBM-positive; 40 CBM-negative) were evaluated. Each POC-LFA was performed in duplicate per sample, generating 100 individual results per assay (20 from CBM-positive sera; 80 from CBM-negative sera).

All positive samples were obtained from culture-positive and CBM-positive dogs. Culture was performed on blood in 8 dogs and on reproductive tract samples (prostate aspirate or vaginal swab) in 2 dogs. Reported clinical presentations included discospondylitis (3 dogs), abortion (1 dog) and a chronic relapsing syndrome characterized by flaccid tail, shifting lameness and pain (1 dog). Two dogs were asymptomatic (identified through screening) and 3 had no clinical history available. Negative control samples were obtained from clinically healthy dogs undergoing pre-export screening, all of which were negative on CBM, 2ME-RSAT, and AGID II.

The Anigen Rapid C.Brucella Ab assay correctly classified 9 out of 10 CBM-positive dogs and 40 out of 40 CBM-negative dogs (Table 1), with a PPA of 90.0% (95% CI, 55.5-99.8) and NPA of 100% (95% CI, 91.1-100; Table 3). One CBM-positive dog (the oldest sample in the dataset) did not meet the dog level concordance criterion and was classified as negative.

**Table 3:** Diagnostic agreement of 3 commercially available POC-LFAs relative to CBM classification (*n* = 50).

Test assay	Positive percent agreement (PPA)	Negative percent agreement (NPA)	Youden index
Anigen Rapid C.Brucella Ab	90.0% (55.5%-99.8%)	100% (91.1%-100%)	0.90 (0.71-1.00)
Canine Brucella LPS Antibody Rapid Test	0% (0%-30.9%)	97.5% (86.8%-99.9%)	−0.03 (−0.07 to 0.02)
FASTest® *BRUCELLA canis*	100% (69.1%-100%)	92.5% (79.6%-98.4%)	0.93 (0.84-1.00)

Table 3 summarizes the performance of 3 commercially available POC-LFA kits relative to CBM classification. Values reflect performance in the 50 dogs included in this study (CBM-positive, *n* = 10; CBM-negative, *n* = 40). Youden index = PPA + PPA − 1. Values shown with 95% CI.

Abbreviations: Ab = antibody; LPS = lipopolysaccharide; POC-LFAs = point-of-care lateral flow assays.

The Canine Brucella LPS Antibody Rapid Test assay correctly classified 0 out of 10 CBM-positive dogs and 39 out of 40 CBM-negative dogs (Table 1), with a PPA of 0% (95% CI, 0-30.9) and NPA of 97.5% (95% CI, 86.8-99.9; Table 3).

The FASTest® *BRUCELLA canis* assay correctly classified 10 out of 10 CBM-positive dogs and 37 out of 40 CBM-negative dogs (Table 1), with a PPA of 100% (95% CI, 69.1-100) and NPA of 92.5% (95% CI, 79.6-98.4; Table 3).

Among the culture-confirmed, CBM-positive dogs, occasional weak test bands were observed for the Anigen Rapid C.Brucella Ab and FASTest® *BRUCELLA canis* assays, but these did not result in disagreement between operators regarding final dog-level classification. All CBM-positive dogs tested using the Canine Brucella LPS Antibody Rapid Test were consistently interpreted as negative by both operators.

In the CBM-negative cohort, all Anigen Rapid C.Brucella Ab results were consistently negative. For the Canine Brucella LPS Antibody Rapid Test, a single weak band resulted in initial disagreement between operators and required third-reader adjudication; the final classification was resolved by majority interpretation. For the FASTest® *BRUCELLA canis* assay, occasional weak-positive bands and one clearly positive result were observed in 3 CBM-negative dogs, but operator interpretations were concordant for each affected sample.

Overall inter-reader agreement was almost perfect across all assays (κ = 0.81-1.00). Agreement for the Anigen Rapid C.Brucella Ab and Canine Brucella LPS Antibody Rapid Test assays was perfect (κ = 1.00; 95% CI, 1.00-1.00; Table 4).

**Table 4:** Interrater reliability for the test assays 3 commercially available POC-LFAs tested.

Test assay	Cohen’s kappa coefficient (95% CI)
Anigen Rapid C.Brucella Ab	1 (1-1)
Canine Brucella LPS Antibody Rapid Test	1 (1-1)
FASTest® *BRUCELLA canis*	0.94 (0.86-1)

Table 4 summarizes the Cohen’s kappa coefficient (κ) of all 3 commercially available POC-LFA kits.

Abbreviations: Ab = antibody; LPS = lipopolysaccharide; POC-LFAs = point-of-care lateral flow assays.

## Discussion

We evaluated the diagnostic performance of 3 commercially available POC-LFAs for the detection of *B. canis* antibodies in canine serum using the Cornell University AHDC CBM assay as the reference serologic assay. Diagnosis of *B. canis* infection remains challenging because of the organism’s slow growth, intermittent bacteremia, and variable antibody responses.^1–3^^,^^9^^,^^11–15^ No single test is definitive across all disease stages, but well-validated, rapid POC assays can support timely clinical decision-making, infection control, and public health protection while pursuing definitive diagnosis. The CBM assay is a fluorescent bead-based multiplex immunoassay that incorporates *B. canis*-specific recombinant antigens, including BP26 and Omp31, and is used in combination with 2ME-RSAT and AGID II as the reference serologic panel in the absence of molecular or microbiological proof of the organism.^16–19^ Manufacturer-reported performance metrics for the LFAs tested are difficult to compare directly, because they are derived using different comparative methods (eg, 2ME-RSAT, RT-PCR, or Rose Bengal testing; Table 2). This heterogeneity emphasizes the need for independent evaluations using a consistent comparative reference serologic panel. Culture or PCR were not used in our study because of the low sensitivity of culture, biosafety risks, and variable PCR performance.^12–14^ In many naturally-infected dogs, presentation occurs weeks to months after exposure, when the initial bacteremic phase may have waned and organisms may be sequestered in reproductive or lymphoid tissues, decreasing the diagnostic yield of blood culture and PCR.^8^^,^^9^^,^^12–14^

The Anigen Rapid C.Brucella Ab and FASTest® *BRUCELLA canis* assays demonstrated high diagnostic agreement with the CBM assay, whereas the Canine Brucella LPS Antibody Rapid Test performed poorly, failing to detect any CBM-positive samples. The Anigen Rapid C.Brucella Ab and FASTest® *BRUCELLA canis* assays achieved PPAs of 90% and 100%, respectively, with NPAs above 92%. Although these findings are broadly consistent with previous studies, in our study the isolated discordant result was most likely attributable to sample-related factors or assay variability. In contrast, false-negative results reported elsewhere have been attributed to early infection before seroconversion.^18^^,^^21^

In our cohort, the only discordant results for the Anigen Rapid C.Brucella Ab and FASTest® *BRUCELLA canis* assays involved the same archived serum sample, which was strongly positive on all reference serologic tests (2ME-RSAT, AGID II, and CBM) and culture-positive. Notably, this sample was the oldest in the dataset, suggesting a sample-related explanation rather than an assay-specific performance issue. Although all serologic reference assays were repeated in parallel at the time of LFA testing and remained positive, degradation of antibody quality, or other storage-related factors cannot be excluded. The specific cause of this anomaly remains uncertain. Larger, prospectively collected datasets are required to determine whether similar discrepancies reflect assay variability or are primarily attributable to sample-related factors.

The poor performance of the Canine Brucella LPS Antibody Rapid Test, compared with its manufacturer-claimed sensitivity, may reflect differences in antigen selection, structural variation in rough-type *B. canis* lipopolysaccharide (LPS) or assay reproducibility. Although the exact antigenic components are proprietary, published descriptions of similar LPS-based assays suggest that the use of generic rough-type LPS may inadequately represent *B. canis* epitopes, which differ structurally from the smooth-type antigens of *Brucella suis* and *Brucella melitensis*.^21–23^ In contrast, the Anigen Rapid C.Brucella Ab and FASTest® *BRUCELLA canis* assays appear to use recombinant *B. canis*-specific proteins that are more consistently expressed across different stages of infection, which may account for their improved PPA and overall agreement.^13^^,^^21^ Variability in manufacturing quality may contribute.^24^ Further characterization of the antigenic composition and production quality of these assays would help clarify the reasons for the observed discrepancies and inform future assay optimization. Cross-reactivity with other bacterial or vector-borne infections was not assessed in our study but has been reported for other *Brucella* serological tests.^13^^,^^23^ Broader validation studies incorporating sera from dogs with confirmed alternative infections would better define real-world specificity and minimize the risk of diagnostic misinterpretation. All 3 POC-LFAs tested showed excellent inter-reader agreement (κ = 0.81-1.00), supporting the conclusion that the performance differences were intrinsic to the assays rather than operator-dependent.

Our study was prompted by the growing need to assess the utility of rapid POC-LFAs in the United Kingdom (UK), where *B. canis* prevalence remains low but importation of infected dogs from endemic regions is an increasing concern.^10^^,^^25^ Initial attempts to source sera from UK-confirmed cases were unsuccessful because of limited availability. Consequently, we used rigorously characterized positive and negative sera from Cornell University, providing a robust analytical comparison despite not being UK-derived. The assays evaluated are commercially available and widely used in the UK, which motivated interpretation of the findings in a UK clinical context. Since October 2025, pre-import testing for *B. canis* has been mandatory in the United Kingdom for commercial imports from Romania.^10^ Nonetheless, these POC assays remain valuable for detecting infections in dogs already present in the country, in animals imported through non-commercial routes and in regions without similar import control measures. Given that the estimated prevalence of *B. canis* in the United Kingdom is likely <0.05%, even a test with 99% specificity would have a positive predictive value <5%.^25^ In practical terms, this means that the majority of positive results obtained in a non-endemic, general screening context would be false-positives, emphasizing the importance of a robust pre-test probability assessment by the clinician.^26^ Accordingly, these assays should be reserved for dogs with appropriate clinical suspicion or epidemiological risk factors, and all positive results should be confirmed using reference serology. False-positive diagnoses may lead to unnecessary isolation, euthanasia, or disruption to breeding programs, as well as undue concern for owners and veterinary staff. In low-prevalence settings, even assays with high sensitivity may generate a disproportionate number of false-positive results if specificity is suboptimal. The high NPA and low false-negative rate observed for the Anigen Rapid C.Brucella Ab and FASTest® *BRUCELLA canis* assays in our dataset support their potential utility as rapid screening tools to help exclude infection in appropriate clinical settings.

Our study had several limitations. Sample size was modest, particularly for positive sera, and no formal power calculation was performed, which may have widened CIs for PPA estimates. The exclusive use of US-derived samples reflects the low prevalence of *B. canis* in the UK and limits immediate assessment of assay performance in UK clinical populations. As such, these findings should be interpreted as reflecting the analytical performance of the assays rather than country-specific epidemiology. Serum samples were archived and frozen before testing, and although all reference serologic panels (2ME-RSAT, AGID II, and CBM assay) were repeated in parallel and remained positive, potential effects of sample age or storage on assay performance cannot be fully excluded. All POC assays were performed using serum only. Therefore, the findings may not be generalizable to other sample types such as EDTA whole blood or plasma, which may affect flow characteristics for some assays. Limited clinical information was available for analysis, although most of the culture-positive dogs had documented clinical signs consistent with *B. canis* infection, and the negative samples were derived from clinically healthy dogs undergoing pre-export screening. Negative controls had complete histories up to the point of recruitment. Long-term follow-up was not available, and undetected infection in the negative group cannot be fully excluded. Nevertheless, all negative dogs were clinically normal and negative on multiple reference assays, suggesting a low probability of subclinical infection. Concurrent infectious diseases were not systematically excluded, but the use of clinically healthy dogs undergoing pre-export screening decreases (but does not eliminate) the likelihood of cross-reactive infections. Separate testing centers were used for positive and negative sera because of biosafety restrictions surrounding the handling and transport of *B. canis*—positive material, which also precluded operator blinding. Although blinding might theoretically have been possible with additional precautions, all assays followed identical written protocols using the same kit batches and standardized reading procedures, and the almost perfect inter-reader agreement suggests that any impact of center-related differences or lack of blinding was minimal. The dichotomized study design (using only clearly defined culture-positive dogs and clinically healthy, seronegative controls) limits applicability to real-world clinical populations, where dogs often present with compatible clinical signs but with unknown infection status. Future studies should therefore include prospectively collected sera from such dogs to better reflect typical diagnostic scenarios and further refine test performance estimates. All CBM-positive samples included in our study were obtained from dogs with confirmed *B. canis* infection based on positive bacterial culture and concordant serologic results. However, culture or PCR was not performed contemporaneously for CBM-negative dogs as part of the present diagnostic comparison. Although bacterial culture is considered the reference standard for definitive diagnosis, it is hazardous, invasive, and frequently insensitive in chronic cases. For these reasons, the CBM assay, a validated and clinically trusted diagnostic test provided by Cornell University AHDC, the recognized *B. canis* reference laboratory in the United States, was used as the reference serologic assay. This laboratory is currently the only facility offering the CBM, 2ME-RSAT, and AGID II assays for routine diagnostic testing. Larger, geographically diverse datasets that incorporate both serologic and molecular testing will be required to validate these findings more broadly. Despite these limitations, our study provides valuable analytical comparison data directly relevant to clinical decision-making.

In conclusion, the Anigen Rapid C.Brucella Ab and FASTest® *BRUCELLA canis* assays showed good agreement with the CBM reference method in this small dataset and may represent rapid, reliable tools to support front-line clinical decision-making in appropriately selected dogs. In contrast, the Canine Brucella LPS Antibody Rapid Test demonstrated unacceptably poor PPA and should not be used for clinical decision-making or as a screening test. In low prevalence settings, these assays are best applied to dogs with compatible clinical signs or epidemiological risk factors, and all positive results require confirmatory reference testing. When used in this context, their high NPA and low false-negative rate support rapid exclusion of infection, facilitating timely patient management, appropriate infection control measures, and protection of veterinary staff and public health.

## Data Availability

Data available on request.

## Off label antimicrobial declaration

Authors declare no off-label use of antimicrobials.

## Institutional animal care and use committee or other approval declaration

Ethical approval was obtained from the University of Edinburgh Veterinary Ethical Review Committee (VERC reference: 142.23).

## Abbreviations

Ab: antibody
AGID: agar gel immunodiffusion test
AGID II: agar gel immunodiffusion test using cytoplasmic antigen
AHDC: Animal Health Diagnostic Center
*B. canis*: Brucella canis
C: control line
CBM: Canine Brucella Multiplex
CI: confidence interval
EDTA: ethylenediaminetetraacetic acid
LFA(s): lateral flow assay(s)
LPS: lipopolysaccharide
mins: minutes
n: number
NPA: negative percent agreement
Omp31: outer membrane protein 31
PCR: polymerase chain reaction
POC: point-of-care
POC-LFA(s): point-of-care lateral flow assay(s)
PPV: positive percent agreement
RT-PCR: reverse transcription-polymerase chain reaction
T: test line
UK: United Kingdom
USA: United States of America
2ME-RSAT: 2-mercaptoethanol rapid slide agglutination test
κ: Cohen’s kappa coefficient
